# Comparative Evaluation of the Antioxidant Capacities of Commonly Consumed Dietary Polyphenols

**DOI:** 10.3390/molecules31172942

**Published:** 2026-08-22

**Authors:** Sevgi Kolaylı, Yakup Kara, Segueni Narimane

**Affiliations:** 1 Department of Chemistry, Faculty of Science, Karadeniz Technical University, 61080 Trabzon, Türkiye; yakupkara@ktu.edu.tr; 2 Department of Biochemistry, Faculty of Medicine, Nakhchivan State University, AZ 7012 Nakhchivan, Azerbaijan; 3 Bee and Bee Products Application and Research Center, Karadeniz Technical University, 61080 Trabzon, Türkiye; 4 Faculty of Medicine, University Salah Boubnider Constantine 3, Constantine 25000, Algeria; segueni_narimane@umc.edu.dz; 5 Laboratory of Naturals Products and Organic Synthesis, Department of Chemistry, Faculty of Exact Sciences, Campus Chaabat Ersas, University Mentouri-Constantine 1, Constantine 25000, Algeria

**Keywords:** antioxidant, polyphenol, FRAP, DPPH, structure

## Abstract

Dietary polyphenols are plant-derived secondary metabolites recognized as highly bioactive molecules. One of their most significant biological properties is their antioxidant potential. In the present study, the antioxidant capacities of commonly consumed dietary polyphenolic compounds were comparatively evaluated. Antioxidant activities were assessed using sing ferric reducing antioxidant power (FRAP) and 2,2-diphenyl-1-picrylhydrazy (DPPH) radical scavenging assays. Twenty-three polyphenol standards were classified into two primary groups, phenolic acids and flavonoids, each further divided into their respective subclasses, and their antioxidant capacities were examined in relation to structural features. Among the phenolic acids, gallic acid (11.29 ± 0.05 µmol Trolox equivalents/mg), protocatechuic acid (6.69 ± 0.04), syringic acid (5.76 ± 0.03), caffeic acid (4.76 ± 0.02), and ellagic acid (4.65 ± 0.07) exhibited the highest FRAP activities. Among the flavonoids, quercetin (5.98 ± 0.03), epicatechin (2.69 ± 0.02), and resveratrol (2.59 ± 0.01) were the most potent antioxidants. Compounds such as chlorogenic acid, ferulic acid, caffeic acid phenethyl ester (CAPE), rutin, and luteolin displayed moderate antioxidant activity, whereas *p*-hydroxybenzoic acid, pinocembrin, chrysin, daidzein, hesperetin, *t*-cinnamic acid, and myricetin showed markedly low activity. These results indicate that antioxidant capacity of polyphenols is strongly influenced by both the number and the positions of hydroxyl groups on their aromatic rings, highlighting the critical role of structural features in determining their redox potential.

## 1. Introduction

Dietary polyphenols are defined specifically as the subclass of plant-derived secondary metabolites containing multiple hydroxyl groups attached to aromatic rings that are ingested as regular, bioactive constituents of the human diet. Based on their structural characteristics, they are classified into different categories, primarily phenolic acids, flavonoids, stilbenes, and lignans. Importantly, dietary polyphenols differ fundamentally from other non-dietary classes of polyphenols—such as structural wood lignins, highly polymerized inedible condensed tannins, industrial tanning agents, or synthetic phenolic compounds. Unlike these non-dietary classes, dietary polyphenols are characterized by their natural occurrence in common food matrices, relatively low molecular weights (typically under 3000 Da), solubility, and biological safety and bioavailability within the human gastrointestinal tract. In plants, polyphenols play a significant role in defense mechanisms against pathogen attacks, ultraviolet (UV) radiation, and various abiotic stress factors [1,2].

In human nutrition, polyphenols are considered powerful antioxidant compounds due to their ability to neutralize free radicals, primarily through electron transfer (ET) and hydrogen atom transfer (HAT) mechanisms, and their ability to chelate metal ions. In addition to their antioxidant activity, they are also associated with anti-inflammatory, antimicrobial, cardioprotective, and immunomodulatory effects. These biological effects are increasingly highlighting the importance of polyphenols in the fields of functional foods and nutraceutical products [3,4].

Foods rich in polyphenols are important sources of functional components from the perspective of food chemistry and nutritional science. Many plant-based foods, primarily bee products such as propolis, honey, pollen, and bee bread, as well as fruits, vegetables, tea, coffee, and whole grains, contain high levels of phenolic acids, flavonoids, and other polyphenolic compounds [5,6]. These compounds significantly contribute to the antioxidant capacity of foods, reducing oxidative stress and modulating inflammatory pathways, though their in vivo bioavailability remains highly dependent on matrix structures and digestive interactions [5,7]. The quantitative levels and profiles of these polyphenols in natural matrices vary significantly based on species, genotype, growing conditions, and harvest times [8,9]. In natural products, a higher concentration of these compounds is often visually associated with darker color tones and environmental stability. For instance, the superior antioxidant capacity observed in dark-colored honeys, such as chestnut or oak honey, is directly linked to their elevated total polyphenol content [10,11], where pigment-like flavonoid derivatives and anthocyanins act as primary active redox components [12,13,14].

Polyphenols constitute a very diverse and extensive family, with more than 8000 distinct compounds identified in natural products to date [4]. This vast chemical diversity arises from variations in the basic aromatic structure, degree of hydroxylation, glycosylation, and other substituent modifications. Consequently, while numerous studies report a positive and statistically significant correlation (often exceeding 65%) between total phenolic content (TPC) and total antioxidant capacity (TAC) in various natural products [11,15,16], this correlation often remains non-linear. This discrepancy strongly suggests that individual phenolic compounds do not possess equivalent antioxidant power, and their specific chemical structures dictate their ultimate redox efficiency.

Despite the abundance of literature on structure–activity relationships of individual polyphenols, a critical knowledge gap persists: directly comparing their relative antioxidant capacities remains challenging due to significant methodological variations among published studies, including differences in experimental conditions and analytical approaches. Moreover, antioxidant activity is strongly influenced by the specific chemical structure of individual polyphenols, and structurally different flavonoids and other phenolic compounds may exhibit markedly different antioxidant and even pro-oxidant properties [8]. Consequently, a systematic comparative study conducted under strictly identical and standardized in vitro conditions is necessary to minimize methodological discrepancies and to better distinguish the intrinsic molecular redox properties of individual dietary polyphenols [8,9,10].

The selection of the FRAP assay as a primary analytical tool in this study is based on its chemistry as a predominantly single-electron transfer (SET)-based measure of ferric-reducing capacity [17,18]. While the FRAP assay is performed under acidic conditions (pH 3.6), which represents a non-physiological environment compared with physiological conditions, this standardized medium provides a well-defined chemical environment for comparing the reducing capacities of structurally diverse compounds [17,19]. The assay is relatively simple, rapid, and reproducible, although its results reflect ferric-reducing capacity rather than antioxidant activity through all possible mechanisms and may be influenced by reaction kinetics and the chemical structure of the tested compounds [18,19]. To complement the mechanistic limitations associated with the acidic FRAP conditions, this study pairs the FRAP assay with the DPPH radical-scavenging assay, which is generally performed in organic solvents because of the limited aqueous solubility of the DPPH radical [18,20]. The use of these complementary assays therefore enables comparison of the intrinsic chemical reducing and radical-scavenging properties of the 23 dietary polyphenols under standardized in vitro conditions, while avoiding direct extrapolation of these chemical measurements to physiological antioxidant efficacy [18,20].

To address this critical need, the present study systematically evaluates the antioxidant capacities of 23 widely consumed dietary polyphenols representing major structural subclasses under completely standardized experimental conditions. By employing a dual-calibration approach using both Trolox and ferrous sulfate equivalents across FRAP and DPPH assays, this study aims to establish a unified molecular-redox map linking specific structural features—including hydroxylation degree, methoxy substitution patterns, esterification, and glycosylation—to antioxidant activity. Such a systematic assessment may help clarify why the relationship between TPC and TAC often remains non-linear in complex natural matrices, where different phenolic profiles can contribute differently to the measured antioxidant response [11,16]. Ultimately, this approach provides a standardized molecular reference for interpreting antioxidant capacity in food chemistry and functional food research.

## 2. Results and Discussions

**Figure 1:**
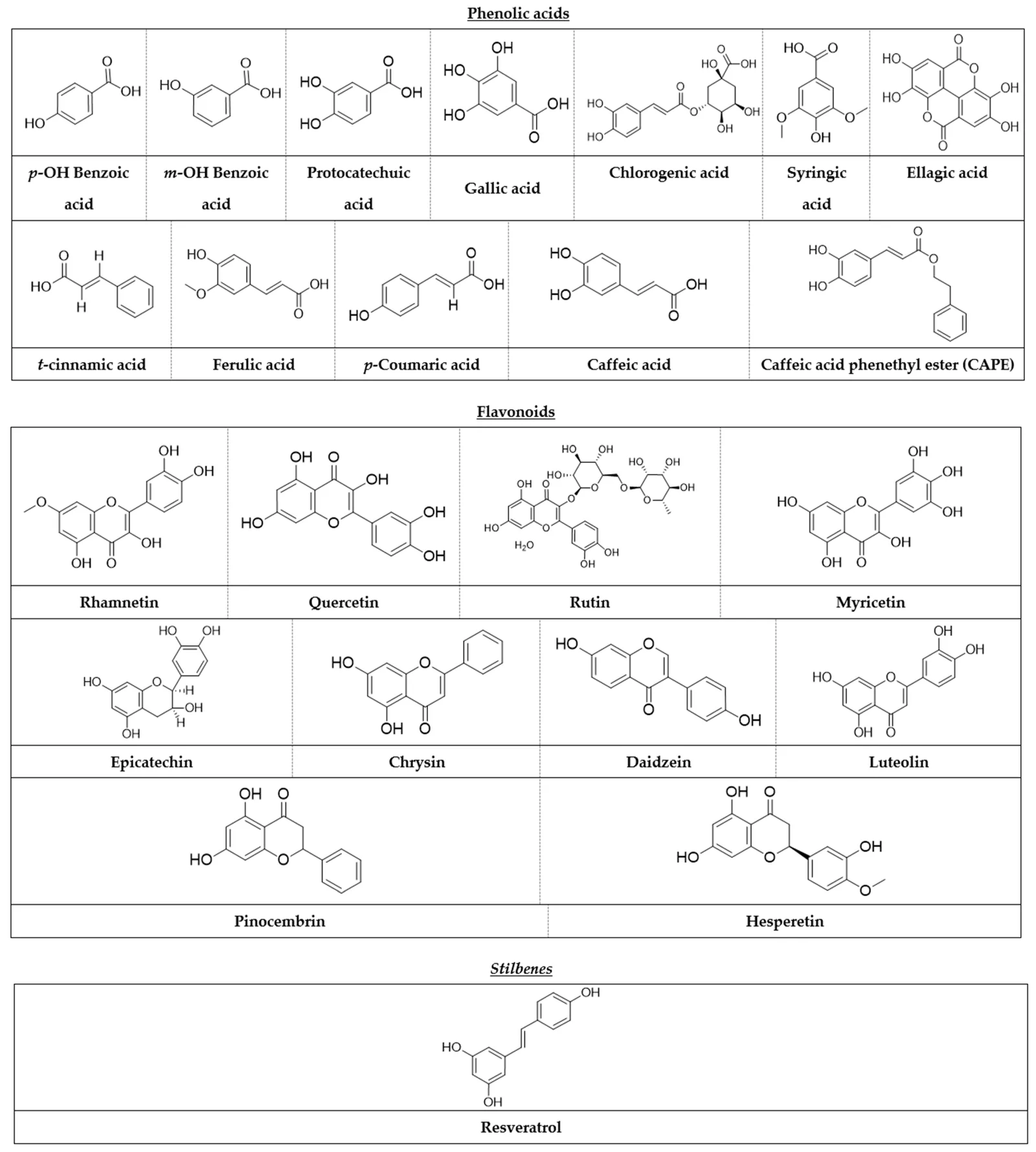
Molecular structures of representative phenolic acids, flavonoids, and stilbenes evaluated in this study.

Table 1 shows the FRAP and DPPH values for the 23 phenolic standards (Figure 1) analyzed in this study. The panel of 23 polyphenolic standards analyzed in this study (Figure 1, Table 1) was strategically selected to represent major dietary subclasses, including phenolic acids (hydroxybenzoic and hydroxycinnamic acids), flavonoids (flavonols, flavan-3-ols, flavones, and flavanones), and stilbenes. These compounds were chosen based on their high dietary abundance and biological relevance, as they are frequently encountered in common foods (fruits, vegetables, tea, coffee) and bioactive functional matrices like bee products (propolis, honey, pollen). By systematically evaluating these highly purified commercial standards (purity) under strictly identical, matrix-free experimental conditions, we successfully isolated their intrinsic redox potentials from the confounding matrix effects and protocol-induced variations typical of crude natural extracts. This standardized design provides a precise molecular map to directly correlate specific structural motifs—such as the degree and position of hydroxylation, methoxy substitution patterns, esterification, and glycosylation—with their corresponding ferric reducing and radical scavenging capacities (Table 2).

These commercially available phenolic compounds were primarily divided into two main groups: phenolic acids and flavonoids. Phenolic acids were further subdivided into hydroxybenzoic acids and hydroxycinnamic acids. Among the seven hydroxybenzoic acid derivatives examined in this study, gallic acid and ellagic acid were found to have the highest reduction capacity values determined by the FRAP method. These findings indicate that these compounds possess strong ferric reduction potential. Under the standardized in vitro parameters of this study, FRAP results are expressed in both Trolox and ferrous sulfate equivalents to ensure methodological robustness (Table 1). A higher FRAP value directly correlates with a superior electron-donating capacity of the pure compound under the acidic conditions of the assay [12,14,21].

Among the seven hydroxybenzoic acid derivatives examined in this study, gallic acid was found to have the highest FRAP value. In contrast, the antioxidant activity of protocatechuic acid and syringic acid was found to be approximately half that of gallic acid. These findings indicate that gallic acid stands out among hydroxybenzoic acids in terms of reducing capacity. When the FRAP values of five different hydroxycinnamic acid derivative phenolic compounds examined in this study were evaluated, caffeic acid was determined to have the highest reducing capacity [22,23]. Caffeic acid was followed by ferulic acid and CAPE, respectively. These results indicate that caffeic acid has a more significant potential in terms of antioxidant power among hydroxycinnamic acid derivatives. Furthermore, it was determined that *p*-hydroxybenzoic acid and *m*-hydroxybenzoic acid compounds had the lowest FRAP values among the phenolic acids studied. This indicates that these molecules have limited ferric ion reducing capacity and therefore exhibit lower antioxidant potential compared to other phenolic acids. Although these two hydroxybenzoic acids are positional isomers of each other (*p*- and *m*-hydroxybenzoic acid), while a low level of reducing activity was measured for *p*-hydroxybenzoic acid in FRAP analysis, no significant FRAP value was detected for *m*-hydroxybenzoic acid. This suggests that the position of the hydroxyl group in the benzene ring may have a decisive effect on the molecule’s electron-donating capacity and, consequently, its ferric ion reduction potential. When the 14 phenolic acid standards used in the study were compared in terms of FRAP activities, gallic acid and syringic acid were determined to have the highest reducing capacity. These findings indicate that these compounds exhibit a higher antioxidant potential compared to other phenolic acids in terms of ferric ion reducing power.

One of the key structural factors determining antioxidant capacity is the number and position of phenolic hydroxyl (-OH) groups in the molecule. As the number of hydroxyl groups increases, the molecule’s thermodynamic ability to donate electrons and reduce metal ions systematically rises [24]. This is exemplified by gallic acid (3,4,5-trihydroxybenzoic acid), which exhibited the highest antioxidant capacity in this study due to its trihydroxylated ring. In contrast, protocatechuic acid contains two hydroxyl groups, while *p*-hydroxybenzoic acid possesses only one, directly explaining their stepwise reduction in activity [24]. The position and electronic properties of substituents further modulate this behavior. Protocatechuic acid features an ortho-dihydroxyl (catechol) system [25], which facilitates the resonance stabilization of the resulting phenoxyl radical, thereby enhancing both HAT and SET pathways [3,26]. Conversely, in syringic acid (4-hydroxy-3,5-dimethoxybenzoic acid), two of these hydroxyl sites are replaced by methoxy (-OCH_3_) groups. While these methoxy groups act as strong electron-donating substituents that transfer electron density to the aromatic ring via resonance (+M) and lower the bond dissociation enthalpy of the remaining OH group [27], the reduction in free hydroxyl stoichiometry ultimately limits its total reducing capacity compared to protocatechuic acid [26,28]. The importance of phenolic hydroxyl groups for redox activity is further illustrated by trans-cinnamic acid; lacking phenolic hydroxyl substituents on its aromatic ring, its reducing and radical scavenging capacities were practically negligible (Table 2) [29].

Significant differences were found between the antioxidant properties of caffeic acid and CAPE. CAPE is a naturally occurring phenethyl ester derivative of caffeic acid, and both compounds are among the main bioactive phenolic components of propolis [30,31]. In the aqueous-buffered FRAP system, free caffeic acid (4.76 ± 0.02 µmol Trolox E./mg) exhibited nearly double the ferric reducing capacity of CAPE (3.01 ± 0.01 µmol Trolox E./mg). Rather than a simple reduction in hydroxyl accessibility, this behavior reflects the electronic influence of esterification on the redox transition states; esterifying the carboxylic acid group alters the conjugated system’s electron density, thereby increasing the ionization potential of the phenolic hydroxyl groups and limiting single-electron transfer pathways. In the literature, it was reported that the free form of caffeic acid generally exhibits higher radical scavenging activity in vitro systems compared to its esterified or more complex derivatives. This is attributed to the electron-withdrawing effect of the carboxylic acid group modulating the redox potential of the molecule and the faster radical scavenging kinetics due to the higher molecular mobility of the free form [32,33]. Interestingly, our DPPH results in organic media revealed the opposite trend: CAPE exhibited a significantly lower (1.48 ± 0.06 µg/mL) value compared to caffeic acid (2.27 ± 0.08 µg/mL). This divergence suggests that the lipophilic phenethyl group enhances radical scavenging kinetics in organic solvents by facilitating hydrogen atom transfer mechanisms, driven by favorable solvent-solute partitioning and resonance stabilization of the transient phenoxyl radical. Therefore, it can be argued that structural modifications occurring through esterification do not diminish antioxidant capacity in an absolute manner; instead, they selectively modulate the kinetic and thermodynamic feasibility of specific electron- or hydrogen-donor pathways depending on the reaction matrix. This finding supports the idea that the structure-activity relationship in phenolic compounds depends not only on the number of hydroxyl groups but also on the chemical nature of the functional groups and the molecular configuration.

Chlorogenic acid is a phenolic compound formed by the esterification of caffeic acid, a derivative of hydroxycinnamic acid, and quinic acid. It exists primarily as 5-O-caffeoylquinic acid. Widely synthesized in plants, this compound is found in high concentrations, particularly in the seeds of Coffea arabica, as well as in apples, pears, and potatoes [34]. Chlorogenic acid was found to have a similar level of DPPH radical scavenging activity to syringic acid. However, its FRAP value, reflecting its ferric-reducing power, was approximately half that of syringic acid. This result indicates that the two compounds exhibit varying performance based on different antioxidant mechanisms. While the DPPH test primarily measures free radical scavenging capacity, the FRAP test evaluates the conversion of the Fe^3+^–TPTZ complex to the Fe^2+^ form based on its reducing potential. Therefore, although chlorogenic acid’s radical neutralization capacity is similar to syringic acid’s, its lower ferric ion reducing capacity may be due to differences in redox potential and electron transfer kinetics [4,32].

**Figure 2:**
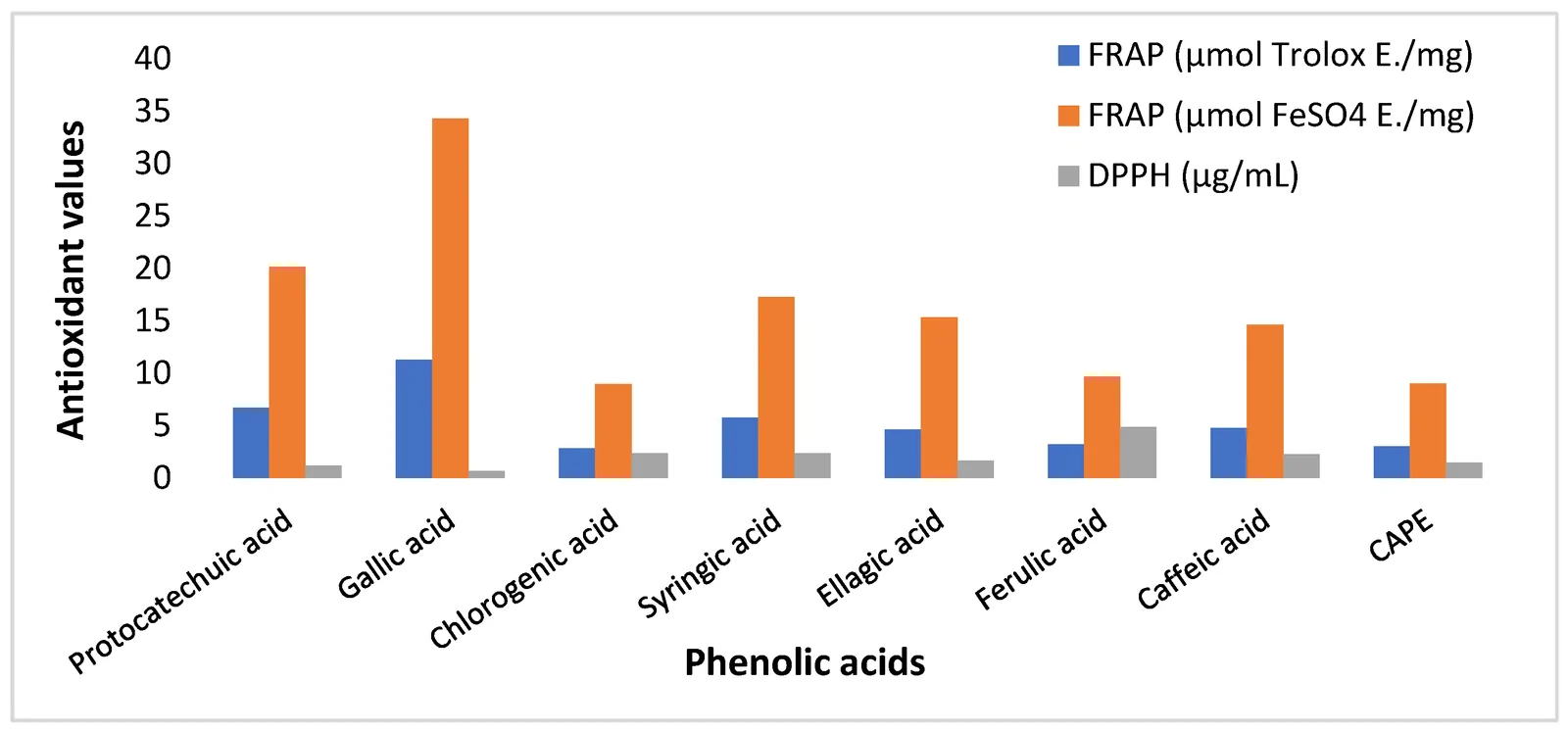
Ferric reducing antioxidant power (FRAP) and DPPH radical scavenging activity of phenolic acid standards. FRAP values are expressed as µmol Trolox E./mg and µmol FeSO_4_ E./mg; DPPH radical scavenging activity is expressed as (µg/mL).

Both ferulic acid and *p*-coumaric acid are derivatives of hydroxycinnamic acid, but the significant difference in their antioxidant activities stems from the additional methoxy (–OCH_3_) group present in the structure of ferulic acid. In ferulic acid, the methoxy group located in the aromatic ring acts as a strong electron-donating group (EDG) with resonance (+M). It transfers electron density to the benzene ring via unpaired electron pairs on the oxygen atom, thus increasing the total electron density of the phenolic system. This facilitates hydrogen atom transfer by lowering the bond dissociation enthalpy of the O–H bond in the adjacent phenolic –OH group. At the same time, the resulting phenoxyl radical becomes more stable due to extended resonance stabilization. In contrast, *p*-coumaric acid contains only a single phenolic hydroxyl group and lacks an additional electron-donating substituent [35]. Therefore, higher energy is required to cleave the phenolic O–H bond, and the resonance stabilization of the resulting radical is more limited. Consequently, the higher antioxidant capacity of ferulic acid compared to *p*-coumaric acid can be explained by the fact that the methoxy group both facilitates the removal of the phenolic hydrogen and stabilizes the resulting radical species. This finding demonstrates that the electronic properties of substituents in phenolic compounds play a decisive role in antioxidant activity [36,37]. While the precise numerical data and statistical groupings are detailed in Table 1, the comparative antioxidant activity trends of the investigated phenolic acids are visually summarized in Figure 2. Comparative evaluation of the results obtained from both antioxidant assay methods demonstrated that gallic acid exhibited the highest antioxidant capacity among the phenolic acids analyzed. This observation suggests that gallic acid possesses superior electron-donating ability and free radical scavenging efficiency relative to the other phenolic acids tested, likely attributable to its structural features, particularly the presence of multiple hydroxyl groups in its aromatic ring.

Flavonoids constitute the largest and most structurally diverse subclass of polyphenols. They are characterized by a common C_6_–C_3_–C_6_ backbone, consisting of two phenyl rings linked by a three-carbon bridge that typically forms a heterocyclic ring [38]. Owing to their well-documented antioxidant properties, flavonoids exhibit significant protective effects on human health, primarily through radical scavenging activity, metal-chelating capacity, and modulation of redox-related biological pathways [7]. In this study, the antioxidant activities of a total of 11 flavonoid standard compounds were compared. The compounds examined were classified into five subgroups according to their structural properties: flavanols, flavan-3-ols, flavones, flavanones, and stilbenes. The results showed that the flavonoids, in particular, possess significant antioxidant potential. Within this group, quercetin exhibited the highest FRAP value and also had the lowest DPPH SC_50_ value. The low SC_50_ value indicates that quercetin has a strong radical neutralization capacity, as it achieves 50% radical scavenging efficiency at lower concentrations. These findings support the idea that the antioxidant activity of flavonoids depends on their structural properties, particularly the degree and position of hydroxylation, and suggest that flavonols may exhibit higher redox activity compared to other flavonoid subgroups, as well as phenolic acids [34].

**Figure 3:**
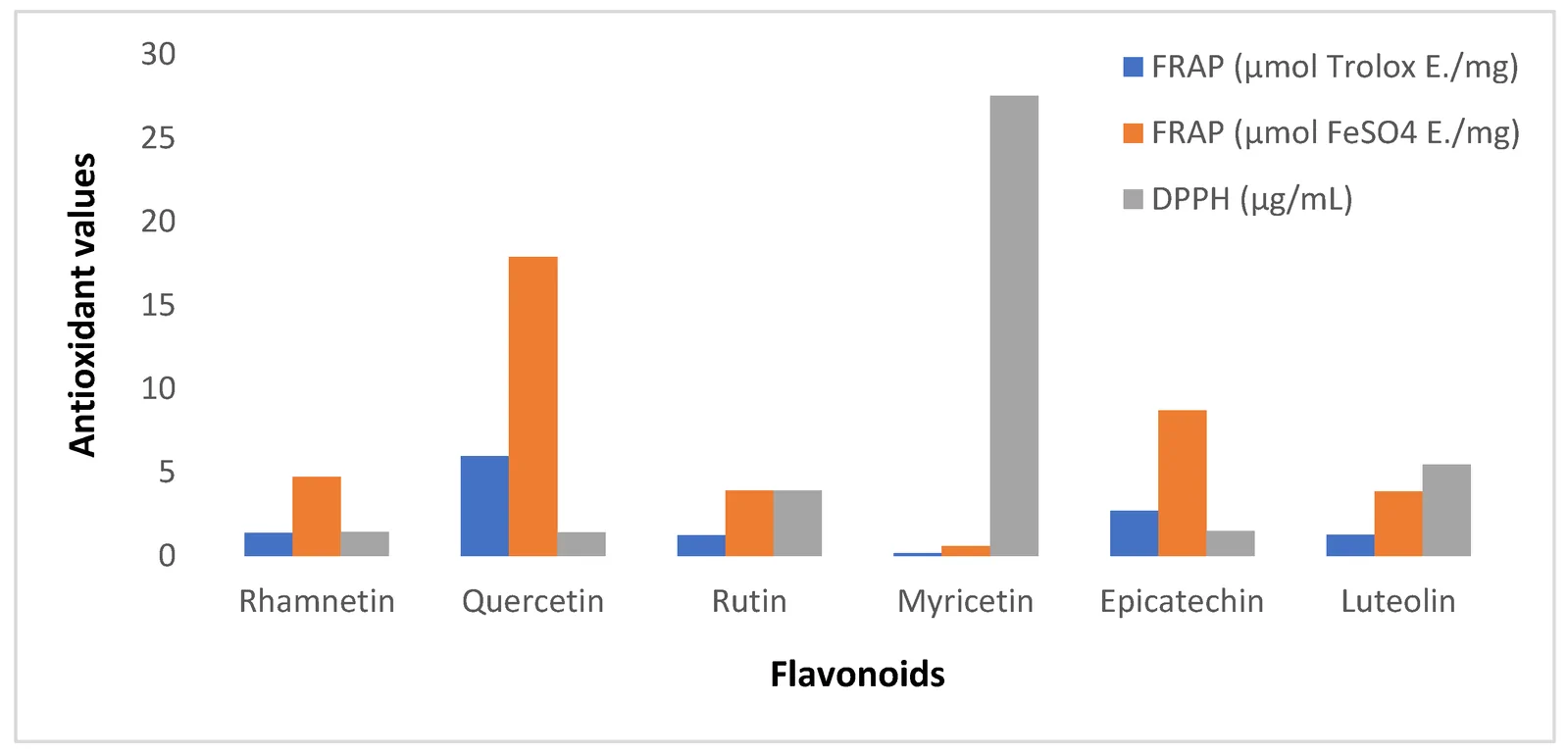
Ferric reducing antioxidant power (FRAP) and DPPH radical scavenging activity of flavonoids standards. FRAP values are expressed as µmol Trolox EE/mg and µmol FeSO_4_ E./mg; DPPH activity is expressed as (µg/mL).

Similarly, Figure 3 visually illustrates the comparative antioxidant profiles of the flavonoid and stilbene subclasses, highlighting the relative activity variations across different structural configurations. Based on the FRAP and DPPH assay results, quercetin demonstrated the highest antioxidant capacity. This finding indicates that quercetin possesses a superior electron-donating capacity, which may be attributed to its structural characteristics, including the presence of multiple hydroxyl groups and an extended conjugated system that enhances redox stabilization. Although quercetin and rutin are similar dietary polyphenols, rutin was found to have much lower DPPH scavenging ability and Fe (III) reduction potential than quercetin. Quercetin is the aglycone form of rutin without a sugar group (rutinose) attached. Due to the free 3-OH group in its molecular structure and the absence of steric hindrance, it exhibits stronger antioxidant activity compared to rutin, its glycoside form [10].

It is known that the rutinose group, a bulky disaccharide in the rutin molecule, forms a steric barrier to the reactive phenolic hydroxyl sites in the aglycone portion, reducing the accessibility of positions that play a critical role in free radical interaction. Due to this steric restriction and the change in electron density distribution, the in vitro antioxidant capacity of rutin is generally lower compared to its aglycone form, quercetin. However, rutin can undergo enzymatic hydrolysis in the gastrointestinal system to convert to its aglycone form, and the resulting quercetin can exhibit strong antioxidant activity in a biological environment. Therefore, when evaluated in terms of dietary intake, it can be said that the difference in antioxidant activity between rutin and quercetin is largely balanced under physiological conditions and is practically negligible [39,40].

Myricetin, a flavonol structurally similar to quercetin, showed unexpectedly low antioxidant capacity in our assays, with low reducing power (FRAP: 0.17 ± 0.00 µmol Trolox E./mg) and a high DPPH SC_50_ value (27.54 ± 0.87 µg/mL). Although myricetin contains a 3′,4′,5′-trihydroxy (pyrogallol) B-ring, compared with the 3′,4′-dihydroxy (catechol) B-ring of quercetin, which would theoretically favor greater electron donation and radical scavenging [41,42,43], antioxidant activity is also strongly influenced by molecular conformation, reaction kinetics, and chemical stability under assay conditions [42]. Pyrogallol-containing phenolics are particularly susceptible to oxygen-mediated autoxidation, with their oxidation behavior being influenced by factors such as pH and oxygen availability [44,45]. Myricetin has also been reported to undergo degradation in solution, with its stability depending on the chemical environment and buffer conditions [46]. Although the methanolic stock solution was introduced into the aqueous FRAP buffer, the substantial dilution of the stock in the final reaction mixture would be expected to minimize concerns regarding incomplete dissolution [41,46]. Thus, the antioxidant activity of myricetin should be interpreted as an assay-dependent response rather than solely on the basis of its degree of hydroxylation [42].

Among the eleven flavonoids evaluated, epicatechin exhibited the second-highest antioxidant capacity after quercetin, followed by resveratrol. Epicatechin is a flavonoid of the flavan-3-ol subclass and a chiral molecule that differs from its trans-isomer, catechin, by its cis configuration (2R,3R). Its high activity is primarily governed by the presence of a catechol moiety (3′,4′-dihydroxy structure) in the B-ring, which promotes rapid radical stabilization via the same resonance mechanisms discussed for protocatechuic acid [47].

Among the three flavone-group compounds evaluated, chrysin, luteolin and daidzein, luteolin exhibited the highest antioxidant capacity under the applied experimental conditions. This superior activity is similarly driven by its B-ring catechol structure, which stabilizes the generated phenoxyl radical through an ortho-quinone-like delocalization and intramolecular hydrogen bonding, thereby facilitating hydrogen atom donation at low bond dissociation energy [48,49].

In flavonoids, the B ring is generally considered the most critical structural element governing antioxidant activity. In the case of luteolin (3′,4′,5,7-tetrahydroxyflavone), its relatively high antioxidant capacity is primarily attributed to the presence of an ortho-dihydroxyl (catechol) structure on the B ring [25]. This catechol moiety enhances radical scavenging efficiency by facilitating hydrogen atom donation at relatively low bond dissociation energy. Following hydrogen transfer, the resulting phenoxyl radical is effectively stabilized through resonance delocalization and intramolecular hydrogen bonding, often involving the formation of an o-quinone-like structure. Such structural stabilization significantly contributes to the overall antioxidant potential of luteolin [3,37].

Chrysin (5,7-dihydroxyflavone) lacks hydroxyl substitution on the B ring and contains hydroxyl groups only on the A ring. The absence of a catechol or other hydroxylated structure in the B ring limits its electron-donating ability and weakens resonance stabilization of the resulting phenoxyl radical. Consequently, chrysin exhibits relatively low antioxidant activity compared with flavonoids bearing ortho-dihydroxylated B-ring systems. In contrast, daidzein, an isoflavone, possesses a single hydroxyl group at the 4′ position of the B ring [50]. The presence of this hydroxyl substituent enhances its radical scavenging and reducing capacity compared with chrysin. However, because daidzein lacks the ortho-dihydroxyl (catechol) configuration found in luteolin, its ability to stabilize the generated radical species is more limited. Therefore, daidzein demonstrates higher antioxidant activity than chrysin, but lower activity than luteolin under comparable experimental conditions.

Literature reports indicate that chrysin is widely distributed in various plant-derived foods and is also frequently detected in bee products such as honey, pollen, propolis, and bee bread [26,51]. Although its antioxidant capacity is comparatively low relative to other flavonoids, chrysin is recognized as a biologically active compound. However, its full spectrum of biological effects and the precise mechanisms underlying its activity have not yet been comprehensively elucidated and remain subjects of ongoing research.

Among the two flavanones evaluated, pinocembrin and hesperetin exhibited markedly different antioxidant capacities. According to the applied assays, pinocembrin demonstrated very weak antioxidant activity compared with hesperetin. This difference can be primarily attributed to variations in B-ring hydroxylation. Pinocembrin lacks hydroxyl substitution on the B ring, which limits its electron-donating capacity and reduces stabilization of the resulting phenoxyl radical. In contrast, hesperetin contains a hydroxyl group on the B ring, enhancing its reducing power and radical scavenging potential. Therefore, the observed disparity in antioxidant activity between these two flavanones can be largely explained by the number and positioning of hydroxyl groups on the B ring. Pinocembrin, similar to chrysin, is a flavanone derivative that is widely distributed in bee products, particularly in propolis, as well as in honey and other hive-derived materials. Its frequent occurrence in these matrices makes it one of the characteristic flavonoid constituents of bee products [52]. The most critical pharmacological feature distinguishing pinocembrin from many other flavonoids is its relatively high lipophilicity, which enables it to effectively cross the blood–brain barrier and reach the central nervous system. This property allows pinocembrin to exert direct neuroprotective effects within brain tissue [53].

Resveratrol was the only stilbene compound evaluated in this study and is recognized as one of the predominant polyphenols in grape seeds [52,54]. In the applied antioxidant assays, resveratrol exhibited a relatively low SC_50_ value in the DPPH radical scavenging test compared with many phenolic acids and flavanols, indicating strong radical scavenging efficiency. Consistently, it also demonstrated a high ferric reducing antioxidant power reflecting its substantial reducing capacity under the tested conditions. The high antioxidant capacity of resveratrol is primarily attributed to the reactivity of its 4′-hydroxyl group and the electron-delocalizing properties of its stilbene backbone. The 4′-OH group readily donates a hydrogen atom or electron to neutralize reactive oxygen species, forming a relatively stable phenoxyl radical. This radical stabilization is facilitated by resonance delocalization across the conjugated double-bond system of the stilbene structure, which enhances electron distribution and reduces the overall redox potential [55]. Consequently, the combined effect of hydroxyl group reactivity and structural electron delocalization underlies the strong antioxidant activity of resveratrol.

To evaluate the statistical consistency of the experimental data and explore the relationship between the underlying chemical mechanisms, Pearson (*r*) and Spearman (ρ) correlation analyses were conducted across the polyphenolic standards. An exceptionally high, statistically significant positive correlation was observed between the two FRAP calibration indices, Trolox and ferrous sulfate equivalents (*r* = 0.999, ρ = 0.997). This confirms that the dual-calibration approach provides highly consistent and reproducible relative rankings of the reducing potentials of dietary phenolics. Furthermore, because the DPPH SC_50_ values spanned several orders of magnitude (ranging from 0.67 to 12,716 µg/mL), these values were log_10_-transformed prior to correlation analysis. A strong and highly significant negative correlation was established between the FRAP values and log_10_(DPPH (SC_50_) values (Pearson r = −0.695, Spearman ρ = −0.886, for FeSO_4_ equivalents). The negative correlation coefficient is chemically consistent with the principles of both assays; as the single-electron transfer-based reducing capacity of a compound increases, the concentration required to scavenge 50% of DPPH free radicals (SC_50_) systematically decreases. The lower Pearson correlation coefficient relative to the Spearman value highlights a slight departure from linearity, primarily driven by the high sensitivity of Pearson’s linear coefficient to extreme statistical outliers in the dataset, such as the exceptionally high values of the inactive compounds. Since Spearman’s rank correlation is robust to such extreme values, it provides a more reliable representation of the overall monotonic relationship. Additionally, minor contributions to this variance may stem from fundamental mechanistic differences, as FRAP operates via single-electron transfer under acidic aqueous conditions, while DPPH involves a combination of pathways in organic solvent systems. Nevertheless, the highly robust Spearman correlation demonstrates that despite these assay-specific and solvent-dependent variations, the structural features of these dietary polyphenols modulate both thermodynamic reducing capacity and kinetic radical scavenging pathways in a highly coordinated and predictable manner.

In addition to electron and hydrogen atom transfer, dietary polyphenols can exert antioxidant effects through the chelation of transition metals such as Fe^3+^, Fe^2+^, and Cu^2+^ [56,57,58]. Catechol and galloyl groups provide effective oxygen-donor sites for metal coordination, while the extent and stability of these interactions depend on the ligand structure and pH [56,57]. This property has different implications for the assays used in the present study. Because DPPH is a metal-free radical probe, metal chelation is not directly measured by this assay; rather, DPPH scavenging primarily reflects electron and/or hydrogen atom transfer processes [18,20]. In contrast, FRAP is based on a Fe^3+^–TPTZ redox system, and differences in the iron-binding properties of polyphenols may influence their apparent ferric-reducing responses under the assay conditions [17,56]. Under physiological conditions, however, transition-metal chelation may contribute to antioxidant protection by limiting the availability of redox-active iron and reducing Fenton-type hydroxyl radical generation [56,58].

From a chemical perspective, it is important to recognize that the acidic conditions of the FRAP assay (pH 3.6) can influence the protonation state and redox behavior of phenolic compounds [17,18]. At this low pH, phenolic hydroxyl groups are predominantly present in their protonated forms, whereas increasing the pH toward physiological conditions can promote partial deprotonation of phenolic groups [59,60]. Such deprotonation may facilitate electron-transfer and proton-coupled electron-transfer processes and thereby alter the apparent antioxidant activity of phenolic compounds [60,61]. Accordingly, the relative antioxidant rankings and absolute reducing values obtained under the standardized acidic FRAP conditions should be interpreted primarily as measurements of ferric-reducing capacity under defined chemical conditions, rather than as direct proxies for antioxidant efficacy under physiological conditions [17,18,60].

Comparing the absolute FRAP and DPPH values obtained in this study with previously published literature reveals certain numerical discrepancies, which highlight the critical need for standardized evaluations. For instance, the reported DPPH SC_50_ values for highly active compounds like gallic acid or quercetin vary significantly across different studies, ranging from minor micromolar levels to much higher concentrations [16,21]. These discrepancies in the literature are primarily attributed to distinct methodological and experimental variations [20,62,63,64]. First, solvent composition plays a key role; varying water-to-solvent (methanol, ethanol, or acetone) ratios can alter DPPH radical-scavenging responses, while solvent and assay conditions can also influence the measured reducing capacity of phenolic compounds [20,63]. Second, reaction incubation periods are not uniformly defined across antioxidant assays, and reaction time can substantially influence the measured radical-scavenging response, particularly for compounds with slower reaction kinetics [63,64]. Third, assay chemistry and reaction conditions, including pH, directly affect the measured antioxidant response because FRAP and DPPH assays operate through different reaction mechanisms and therefore do not necessarily produce identical antioxidant rankings for the same compounds [62,64]. By evaluating all 23 dietary polyphenols under strictly identical and standardized conditions, this study minimizes these protocol-induced confounding factors and provides a unified relative-potency scale [20,62].

Although the combined application of FRAP and DPPH assays provides valuable preliminary insights into the reducing power and radical scavenging capacities of the evaluated polyphenols, these in vitro chemical assays have inherent limitations that should be considered when interpreting their antioxidant potential. First, the FRAP assay is performed under acidic conditions (pH 3.6), which may alter the protonation states, molecular charges, and consequently the redox reactivities of certain polyphenols compared with those under physiological pH conditions. Second, neither assay directly employs biologically representative reactive oxygen species; DPPH is a stable, synthetic organic radical, and these assays do not directly assess scavenging activity against physiologically relevant reactive oxygen species, such as superoxide (O_2_•^−^) or hydroxyl radicals, nor do they reproduce the complexity of lipophilic biological membrane environments.

In summary, the established relative potency rankings of these 23 standards highlight that antioxidant capacity is a multi-mechanistic chemical property heavily dictated by molecular architecture rather than a simple concentration-dependent variable.

## 3. Materials and Methods

### 3.1. Chemicals

All chemicals and reagents used in this study were of analytical grade. Quercetin standard (≥98% purity, Cat. No. 6151-25-3) was obtained from Lancaster Synthesis Ltd. (Morecambe, Lancashire, UK), while other phenolic standards were purchased from Sigma-Aldrich (Schnelldorf, Germany). Glacial acetic acid, TPTZ (2,4,6-tripyridyl-s-triazine), and 2,2-diphenyl-1-picrylhydrazyl (DPPH) were obtained from Sigma-Aldrich (St. Louis, MO, USA). Methanol was supplied by Isolab (Eschau, Germany). Folin–Ciocalteu reagent and FeSO_4_·7H_2_O were purchased from Merck (Darmstadt, Germany), and FeCl_3_ was obtained from Carlo Erba (Val-de-Reuil, France).

### 3.2. Preparation of Phenolic Standard Solutions

Stock solutions of all twenty-three dietary polyphenol standards were prepared in analytical-grade methanol at appropriate concentrations. To minimize light-induced degradation and potential kinetic instability, the stock solutions were prepared fresh, protected from light by wrapping in aluminum foil, and stored at −20 °C until analysis. Working solutions for the FRAP and DPPH assays were prepared immediately before analysis by appropriate serial dilutions of the methanolic stock solutions using analytical-grade methanol [17,18,19,20,21,22,23,24,25,26,27,28,29,30,31,32,33,34,35,36,37,38,39,40,41,42,43,44,45,46,47,48,49,50,51,52,53,54,55,56,57,58,59,60,61,62,63,64,65].

### 3.3. Determination of Ferric Reducing Antioxidant Power (FRAP)

Ferric reducing antioxidant power assay was performed according to the method [17]. The assay is based on the reduction of Fe^3+^–TPTZ complex to the ferrous (Fe^2+^) form in the presence of antioxidant compounds, resulting in the formation of an intense blue Fe^2+^–TPTZ complex with maximum absorbance at 593 nm.

The FRAP reagent was freshly prepared prior to analysis by mixing 300 mM acetate buffer (pH 3.6), 10 mM TPTZ solution, and 20 mM FeCl_3_ solution in a ratio of 10:1:1 (*v*/*v*/*v*). Trolox was used to construct the standard calibration curve. A 1000 μM Trolox stock solution was prepared and serially diluted to obtain working standards. After completion of the reaction, absorbance values were plotted against Trolox concentrations to generate the calibration curve, and the antioxidant capacity of the samples was calculated accordingly (Figure S1). For the Trolox equivalent, a separate calibration curve was prepared using Trolox standards (25–400 µM), and FRAP activity was expressed as µmol Trolox equivalents (TE) per mg of phenolic standard. For the ferrous ion equivalent, a calibration curve was constructed using FeSO_4_·7H_2_O standards (31.25–1000 µM), and the antioxidant capacity was expressed as µmol FeSO_4_ per mg of phenolic standard (Figure S2).

### 3.4. Determination of DPPH Radical Scavenging Activity

The DPPH radical scavenging activity was determined according to the method [66]. DPPH radical, a synthetically produced stable free radical, exhibits maximum absorbance at 517 nm. A 100 μM methanolic DPPH· solution was prepared for the assay. To evaluate the radical scavenging activity of the samples, serially diluted sample solutions were added to a fixed concentration of DPPH· solution. The sample solvent was used as the reagent blank. The reaction mixture, consisting of sample and DPPH· solution at a ratio of 1:1 (*v*/*v*), was incubated under dark conditions, and the decrease in absorbance was measured spectrophotometrically at 517 nm. The radical scavenging activity was calculated from the plotted inhibition curve, and results were expressed as SC_50_ values (the concentration required to scavenge 50% of DPPH· radicals). Trolox was used as a reference standard to compare antioxidant capacities. All measurements were performed in triplicate.

### 3.5. Statistical Analysis

All experimental analyses were performed in triplicate, and data are expressed as the arithmetic mean ± standard deviation (mean ± SD). Basic data processing, calculations, and graphical representations were performed using Microsoft Excel. To determine the statistical significance of the differences among the antioxidant capacities of the 23 dietary polyphenols, a One-way Analysis of Variance (ANOVA) was conducted for each assay. This was followed by Tukey’s HSD post hoc test for pairwise multiple comparisons to identify statistically significant differences between individual compounds and subclasses. Additionally, to evaluate the methodological consistency and explore the relationships between the different antioxidant assays, quantitative bivariate correlation analyses were conducted using both Pearson’s linear correlation (*r*) and Spearman’s rank-order correlation (ρ) coefficients. Due to the wide range of concentration values spanning several orders of magnitude, DPPH (SC_50_) values were log_10_-transformed prior to correlation analysis to ensure statistical robustness. A *p*-value of less than 0.05 (*p* < 0.05) was considered statistically significant, and all advanced statistical computations were performed using SPSS software (version 26.0, IBM Corp., Armonk, NY, USA).

## 4. Conclusions

In this study, the antioxidant capacities of 23 representative standard phenolic molecules spanning key subclasses, including phenolic acids, flavonoids, and stilbenes, were systematically evaluated under strictly standardized experimental conditions. Unlike isolated structure–activity studies relying on heterogeneous methodologies, the use of identical experimental parameters and a robust dual-calibration approach based on Trolox and ferrous sulfate equivalents enabled a direct comparison of the intrinsic redox properties of these compounds while minimizing protocol- and matrix-related confounding factors. The findings demonstrate that phenolic compounds do not possess equivalent antioxidant capacities; rather, their redox activities are strongly influenced by specific structural features, including the number and position of hydroxyl groups, degree of conjugation, redox potential, and substituent properties. Gallic acid and quercetin emerged as the most potent reducing and radical-scavenging agents within their respective subclasses, whereas the importance of aromatic hydroxylation was further demonstrated by the negligible activities of trans-cinnamic acid and chrysin. Furthermore, the study provided experimental evidence for the distinct behavior of highly hydroxylated structures, as illustrated by myricetin, whose pyrogallol-containing B-ring was associated with kinetic autoxidative instability under the assay conditions, thereby compromising its measurable reducing capacity. The comparative analysis of caffeic acid and its esterified derivative, CAPE, further demonstrated that structural modifications such as esterification do not uniformly diminish antioxidant activity but can selectively modulate electron- and hydrogen-transfer pathways depending on molecular and solvent properties.

The robust consistency of the dual-calibration approach between Trolox and FeSO_4_ equivalents (r = 0.999) supports the analytical reliability of the FRAP measurements, whereas the strong but inverse relationship between FRAP and DPPH responses (Spearman ρ = −0.886) highlights the mechanistic differences between electron-transfer- and hydrogen-atom-transfer-dominated antioxidant responses. These findings provide a molecular-level explanation for why TPC does not necessarily correlate linearly with TAC in complex natural matrices, where the qualitative composition and structural characteristics of individual phenolic compounds can contribute differently to the overall antioxidant response. Thus, the standardized molecular-redox profiles generated in this study provide a useful reference framework for food chemists and functional food developers in interpreting and optimizing the antioxidant performance of polyphenol-rich formulations. Nevertheless, the generalizability of these findings is naturally constrained by the selection of 23 standards—constituting a minor fraction of the ~8000 natural polyphenols—and our focus on single-solvent, acidic or organic environments. To build a more comprehensive molecular-redox map, future research should incorporate complementary assays across diverse pH conditions and solvent systems. Utilizing multi-assay frameworks, such as CUPRAC, ABTS, and ORAC, will be crucial to characterize these redox behaviors under varying environments, ultimately bridging the gap between in vitro chemical measurements and in vivo biological effects.

## Figures and Tables

**Table 1:** Antioxidant capacities of dietary polyphenols.

	Polyphenolic Standards	FRAP (µmol Trolox E./mg)	FRAP (µmol FeSO_4_ E./mg)	DPPH (µg/mL)
Phenolic acids	Hydroxybenzoic acids			
	*p*-OH Benzoic acid	-	0.04 ± 0.02 ^o^	11,105.77 ± 62.82 ^n^
	*m*-OH Benzoic acid	-	-	4725.00 ± 31.82 ^m^
	Protocatechuic Acid	6.69 ± 0.04 ^b^	20.13 ± 0.10 ^b^	1.18 ± 0.03 ^b^
	Gallic acid	11.29 ± 0.05 ^a^	34.29 ± 0.15 ^a^	0.67 ± 0.06 ^a^
	Chlorogenic acid	2.85 ± 0.01 ^i^	8.97 ± 0.02 ^h^	2.38 ± 0.04 ^e^
	Syringic acid	5.76 ± 0.03 ^d^	17.27 ± 0.08 ^d^	2.35 ± 0.05 ^e^
	Ellagic acid	4.65 ± 0.07 ^f^	15.32 ± 0.20 ^e^	1.65 ± 0.04 ^d^
	Hydroxycinnamic acids			
	*t*-cinnamic acid	-	-	12,716.67 ± 94.99 ^o^
	Ferulic acid	3.22 ± 0.01 ^g^	9.69 ± 0.03 ^g^	4.87 ± 0.11 ^g^
	*p*-Coumaric acid	0.20 ± 0.00 ^o^	0.60 ± 0.00 ^n^	1569.81 ± 12.63 ^k^
	Caffeic acid	4.76 ± 0.02 ^e^	14.63 ± 0.05 ^f^	2.27 ± 0.08 ^e^
	Caffeic acid phenethyl ester (CAPE)	3.01 ± 0.01 ^h^	9.00 ± 0.03 ^h^	1.48 ± 0.06 ^c,d^
Flavonoids	Flavonols			
	Rhamnetin	1.37 ± 0.02 ^l^	4.73 ± 0.04 ^k^	1.42 ± 0.05 ^c^
	Quercetin	5.98 ± 0.03 ^c^	17.90 ± 0.08 ^c^	1.40 ± 0.03 ^c^
	Rutin	1.23 ± 0.01 ^m^	3.91 ± 0.01 ^l^	3.90 ± 0.09 ^f^
	Myricetin	0.17 ± 0.00 ^o^	0.57 ± 0.01 ^n^	27.54 ± 0.87 ^h^
	Flavan-3-ols			
	Epicatechin	2.69 ± 0.02 ^j^	8.70 ± 0.05 ^i^	1.48 ± 0.07 ^c,d^
	Flavones			
	Chrysin	0.02 ± 0.00 ^p^	0.08 ± 0.00 ^o^	11,992.34 ± 389.29 ^n,o^
	Daidzein	0.03 ± 0.00 ^p^	0.13 ± 0.00 ^o^	418.11 ± 40.85 ^j^
	Luteolin	1.27 ± 0.01 ^m^	3.84 ± 0.02 ^l^	5.45 ± 0.10 ^g^
	Flavanones			
	Pinocembrin	-	0.01 ± 0.00 ^o^	3052.69 ± 28.40 ^l^
	Hesperetin	0.30 ± 0.00 ^n^	0.91 ± 0.01 ^m^	192.56 ± 17.55 ^i^
Non-Flavonoids	Stilbenes			
	Resveratrol	2.59 ± 0.01 ^k^	7.61 ± 0.04 ^j^	5.41 ± 0.10 ^g^
Trolox		*	*	4.00 ± 0.12 ^f^

-: not detected, (*): not measured. Different superscript letters (a–p) in the same column indicate statistically significant differences among the polyphenolic compounds (*p* < 0.05) according to One-way ANOVA followed by Tukey’s post hoc test. Values are expressed as mean ± SD (*n* = 3). SC_50_ values exceeding 1000 µg/mL are mathematically extrapolated from non-linear regression curves due to the low radical-scavenging activity of these compounds.

**Table 2:** Structure–activity relationships (SAR) of the 23 investigated dietary polyphenols under standardized conditions.

Polyphenol Subclass	Key Structural Motifs	Investigated Compounds (All 23 Standards Mapped)	Ferric Reducing Power (FRAP)	Radical Scavenging Potential (DPPH)	Primary SAR Mechanism
Hydroxybenzoic acids (7 compounds)	Trihydroxyl (pyrogallol) ortho-dihydroxyl (catechol) Methoxy + hydroxyl substitution Isolated monohydroxyl	Gallic acid Ellagic acid Protocatechuic acid Syringic acid Chlorogenic acid *p*-OH Benzoic acid *m*-OH Benzoic acid	Gallic acid: Very High Protocatechuic, Syringic, Ellagic, Chlorogenic: High to Moderate *p*-OH, *m*-OH: Low to Negligible	Gallic, Protocatechuic, Ellagic, Chlorogenic, Syringic: Very High to High *p*-OH, *m*-OH: Negligible	Adjacent hydroxyl groups promote strong resonance stabilization of phenoxyl radicals. Isolated mono-hydroxyls (*p*- and *m*-OH benzoic acids) severely restrict hydrogen atom/electron transfer.
Hydroxycinnamic acids (5 compounds)	Conjugated double bond with catechol Methoxy + hydroxyl substitution Phenethyl esterification Monohydroxyl Unsubstituted backbone	8. Caffeic acid 9. Ferulic acid 10. CAPE 11. *p*-Coumaric acid 12. *t*-Cinnamic acid	Caffeic acid: High Ferulic acid, CAPE: Moderate *p*-Coumaric, *t*-Cinnamic: Low to Negligible	CAPE: Very High Caffeic acid: High Ferulic acid: Moderate *p*-Coumaric, *t*-Cinnamic: Low to Negligible	The ethylenic side chain enhances resonance delocalization. Methoxy groups stabilize radicals. Esterification (CAPE) selectively enhances radical scavenging kinetics in organic media (DPPH).
Flavonols (4 compounds)	Catechol B-ring, free 3-OH, 4-oxo system 3-O-glycosylation Unstable trihydroxyl B-ring	13. Quercetin 14. Rhamnetin 15. Rutin 16. Myricetin	Quercetin: Very High Rhamnetin, Rutin: Moderate Myricetin: Negligible	Quercetin: Very High Rhamnetin, Rutin: Moderate to High Myricetin: Low	Quercetin satisfies all major antioxidant criteria. Glycosylation (rutin) or methylation (rhamnetin) introduces steric hindrance. Myricetin suffers from the ‘pyrogallol paradox’ (rapid autoxidation and polymerization).
Flavan-3-ols (1 compound)	Catechol B-ring Saturated C-ring (lacks 2,3-double bond and 4-oxo)	17. Epicatechin	Epicatechin: Moderate to High	Epicatechin: Very High	The saturated C-ring decreases metal-reduction potential, but the active B-ring catechol system retains high radical scavenging capacity.
Flavones & Flavanones (5 compounds)	B-ring catechol system Mono-hydroxyl B-ring Unsubstituted B-ring	18. Luteolin 19. Chrysin 20. Daidzein 21. Hesperetin 22. Pinocembrin	Luteolin: Moderate Hesperetin: Low Chrysin, Daidzein, Pinocembrin: Negligible	Luteolin: Moderate Hesperetin, Daidzein: Moderate to High Chrysin, Pinocembrin: Negligible	B-ring hydroxylation is the primary driver of redox activity. Complete lack of B-ring hydroxyl substituents (chrysin, pinocembrin) virtually abolishes activity.
Stilbenes (1 compound)	Conjugated stilbene backbone Reactive 4′-hydroxyl group	23. Resveratrol	Resveratrol: Moderate	Resveratrol: Moderate to High	The conjugated stilbene double-bond system facilitates extensive delocalization of the transient phenoxyl radical across both rings.

## Data Availability

The data supporting the findings of this study are available from the corresponding author upon reasonable request.

## Supplementary Materials

The following supporting information can be downloaded at: https://www.mdpi.com/article/10.3390/molecules31172942/s1, Figure S1: Trolox standard (400-25 µM) to measure FRAP values; Figure S2: FeSO_4_·7H_2_O standard graph (1000-31.25 µM) to measure FRAP values.

## Abbreviations

The following abbreviations are used in this manuscript:

FRAP	Ferric Reducing Antioxidant Power
TPTZ	2,4,6-tripyridyl-s-triazine
DPPH	2,2-Diphenyl-1-picrylhydrazyl
SC_50_	Radical Scavenging Concentrations
CAPE	Caffeic Acid Phenethyl Ester
CUPRAC	Cupric Ion Reducing Antioxidant Capacity
ABTS	2,2′-Azino-bis(3-ethylbenzothiazoline-6-sulfonic acid)
ORAC	Oxygen Radical Absorbance Capacity
