# Supplementary material for: Comparative Evaluation of the Antioxidant Capacities of Commonly Consumed Dietary Polyphenols
Source: Molecules. 2026 Aug 22;31(17):2942. doi: 10.3390/molecules31172942 (PMC13567273; doi:10.3390/molecules31172942)
Supplement: Supplementary file 1 [file molecules-31-02942-s001.zip › molecules-4497236-supplementary.pdf]

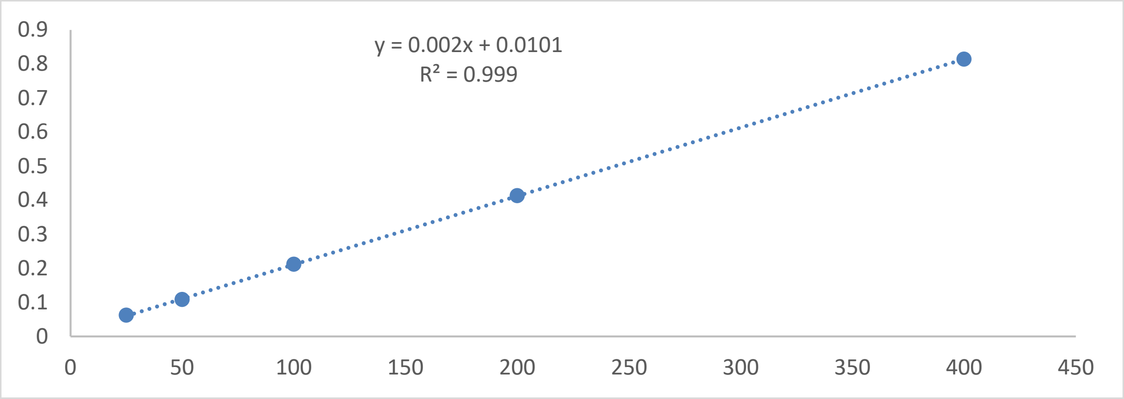

Figure S1. Trolox standard (400-25 μM) to measure FRAP values

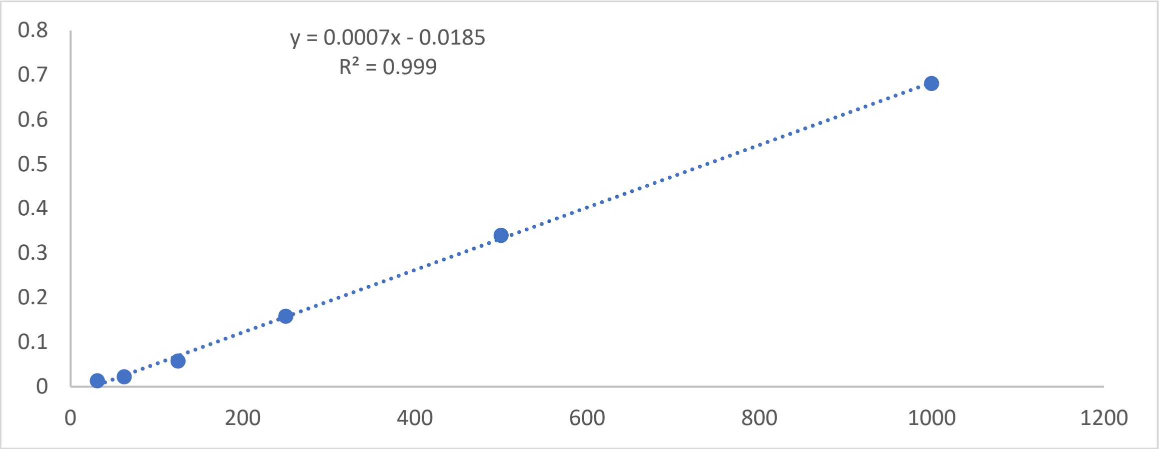

Figure S2. $FeSO_4{\cdot}7H_2O$ standard graph (1000-31.25 μM) to measure FRAP values
